# Peripheral immune cell subsets as potential predictors of benefit from immune checkpoint blockade therapy in small cell lung cancer

**DOI:** 10.3389/fimmu.2026.1802274

**Published:** 2026-06-12

**Authors:** Miguel A. Galindo-Campos, Max Hardy-Werbin, Joan Gibert, Margherita Pucci, Anna Hernández-Prat, Alejandro Ríos-Hoyo, Pedro Rocha, Adriá Rossell, Raúl del Rey-Vergara, Ana Parreira, Andrés Pellicer, Álvaro Taus, Ana Rovira, Edurne Arriola

**Affiliations:** 1 Cancer Research Program, Hospital del Mar Research Institute, Barcelona, Spain; 2 Medical Oncology Department, Hospital del Mar, Barcelona, Spain; 3 Pathology Department, Hospital del Mar, Barcelona, Spain; 4 Medical Oncology Department, The Christie National Health Service (NHS) Foundation Trust, Manchester, United Kingdom; 5 Centro de Investigación Biomédica en Red de Oncología, (CIBERONC), Instituto de Salud Carlos III (ISCIII), Madrid, Spain

**Keywords:** biomarkers, immune checkpoint blockade therapy (ICBt), immunotherapy, peripheral blood mononuclear cells (PBMCs), prognostic predictive factors, small cell lung cancer (SCLC)

## Abstract

**Background:**

Small cell lung cancer (SCLC) is a lethal neoplasia. Chemotherapy (Ct) plus immune checkpoint blockade therapy (ICBt) is now the standard of care although limited survival benefit. Lack of biomarkers of response leads to suboptimal patient selection, distorting results of clinical trials. Deciphering the ICBt-driven peripheral immune events may recognize those patients who benefit the most. Here, we characterize early peripheral immune kinetics in SCLC patients treated with Ct + ICBt with improved survival.

**Methods:**

SCLC patients from 8 centers were prospectively recruited into 3 cohorts. Cohort 1 (N = 24) include patients treated with Ct. Cohort 2 (N = 37) comprise patients treated with Ct + anti-CTLA-4, and cohort 3 (N = 20) patients treated with Ct + anti-PD-1/PD-L1. Peripheral blood mononuclear cells (PBMCs) were obtained prior to treatment initiation and 3–4 weeks after (before cycle 2). Variation in proliferation, senescence, adhesion, immunosuppression, and checkpoints markers was assessed by Fluorescence-Activated Cell Sorting (FACS). Kaplan–Meier and log-rank test were used for survival analysis. MaxStat was used to calculate cut points. A p-value <0.05 was considered statistically significant.

**Results:**

We found 6 independent cellular subsets whose modulation shortly after the start of ICBt identify patients with improved survival. An increase in CD8+CD103+Ki67+ cells identify survival benefit in cohorts 1 and 3 (p=0.043; 0.0033). In ICBt cohorts 2 and 3, an upregulation of Ki67 in total CD4+ (p=0.012; 0.0027) and CD4+PD-1+ T cells (p=0.026; 0.027), predicted longer survival, while a downregulation of CD4+ICOS+ T cells (p=0.025; 0.011) identified survival benefit in these same ICBt cohorts. Expansion of Ki67+ and ICOS+ (p=0.0024; 0.0074) CD8+ T cells was also observed in CD8+ T cells from long survivors exclusively from cohort 2.

**Conclusions:**

This study provides one of the few longitudinal assessments of peripheral immune dynamics in SCLC patients receiving ICBt, suggesting that early on-treatment changes, evaluated relative to each patient’s own baseline levels, may provide greater predictive value than single-timepoint inter-patient comparisons.

## 1 Introduction

Small cell lung cancer (SCLC) is the most lethal subtype of lung cancer and accounts for approximately 15% of all cases (1). The majority of patients are diagnosed with stage IV or extensive disease (ED) and present a 5-year overall survival (OS) of less than 5% (2). Etoposide plus platinum-based chemotherapy has remained unchanged as the standard-of-care for treating SCLC patients until the recent addition of immune checkpoint blockade therapy (ICBt) to frontline treatment, adding a marginal benefit in a subset of patients (3). More recently, addition of lurbinectedin in maintenance has demonstrated an increase in both progression-free survival (PFS) and OS in a subset of these patients (4).

Classically, SCLC is defined as an immune-cold cancer (5). Nevertheless, the recent SCLC transcriptional stratification into four molecular subtypes, revealed that the SCLC-I (inflamed) subtype shows upregulation of proinflammatory genes potentially associated with benefit from addition of ICBt to standard chemotherapy (6–8).

The immune events triggered by the ICBt are not clearly defined in SCLC and their understanding may guide the identification of biomarkers for a precise selection of patients who truly benefit from ICBt. The lack of biopsy material and the rapid deterioration of patients at progression make intra-tumoral biomarkers studies particularly challenging for this disease. Peripheral immune cells might be a potential non-invasive source for biomarkers in a clinical setting of immune modulation. Correlation of circulating immune subsets and survival has been studied in several cancer types treated with ICBt, such as bladder cancer (9, 10), melanoma (11–13), head and neck squamous cell carcinoma (14), non-small cell lung cancer (NSCLC) (15), as well as in a pan-cancer analysis encompassing multiple solid tumor types (16). A 2022 report from members of our group in NSCLC patients provided addition evidence supporting this association (17). Additionally, in a previous study from our group, circulating antineuronal antibodies were identified as prognostic biomarkers in SCLC receiving chemoimmunotherapy compared with chemotherapy alone (18).

In the current work, we explored basal and on-treatment immune biomarkers and their dynamic changes in peripheral blood of 3 cohorts of patients with ED-SCLC treated with different schemes: chemotherapy alone, chemotherapy plus anti-CTLA-4 agent, and chemotherapy plus anti-PD-1/PD-L1 agents. We report specific peripheral immune events that correlate to SCLC patients’ survival differentially when treated with chemotherapy in combination with either anti-CTLA-4 or anti-PD-1/PD-L1 therapies.

Our findings suggest that peripheral immune modulation may be specific to distinct ICBt mechanisms of action. Incorporating both baseline and on-treatment blood sampling in future prospective studies could help identify patients most likely to benefit from each therapeutic strategy.

## 2 Methods

### 2.1 Patient populations

Newly diagnosed patients with stage IV SCLC were prospectively included into 3 cohorts. Patients from cohort 1 (N = 24) were recruited between October 2016 and January 2020 at Hospital del Mar (Barcelona, Spain) and treated with platinum plus etoposide-based chemotherapy. Cohort 2 (N = 37) included patients from 8 different medical centers from the United Kingdom recruited for a multicenter phase II clinical trial [ICE-trial, NCT01331525] who were treated with the anti-CTLA-4 agent ipilimumab in combination with platinum and etoposide, and which detailed results were reported elsewhere (19). Cohort 3 (N = 20) included patients locally recruited at Hospital del Mar (Barcelona, Spain) between December 2020 and April 2021 who were treated with chemotherapy plus anti-PD-1/PD-L1 agents (pembrolizumab, durvalumab or atezolizumab). Subsequently, a subset of patients underwent additional chemotherapy-based treatments; however, complete information regarding subsequent lines of therapy was not available.

### 2.2 Sample collection

Blood samples were obtained by standard venipuncture into 10 mL ethylenediaminetetraacetic acid (EDTA) Vacutainer tubes (BD Becton Dickinson) prior to treatment (Baseline) and after the first treatment cycle (Timepoint 2) (**Supplementary Table 1**). Timepoint 2 occurred 3–4 weeks after cycle 1, immediately prior to the administration of cycle 2 of platinum plus etoposide-based chemotherapy, anti-CTLA-4 or anti-PD-1/PD-L1 agents. Peripheral blood mononuclear cells (PBMCs) were isolated using density gradient medium (Lymphoprep, Stemcell Technologies) following manufacturer´s protocol and cryopreserved at a density of 8–10 X 10^6^ cells/mL in cryopreservation media composed of 90% fetal bovine serum (FBS) (Sigma-Aldrich) and 10% dimethyl sulfoxide (DMSO) (Sigma-Aldrich). PBMCs were then stored for 24 hours at -80°C and afterwards at -190°C in liquid nitrogen for long term storage until thawing and staining.

Serum samples from patients in cohort 2 were received already processed and stored at -80°C at the time of the previously published study (20), during which cytokine measurements were performed, including Th1 (IFN-γ, IL-2, TNF-α), Th2 (IL-4, IL-5, IL-10), and inflammatory cytokines (GM-CSF, IL-1β, IL-6, IL-8), as well as MIP-1α. No additional cytokine analyses were performed in the present study.

Sample and data collection were approved by the local ethics committee of the participating institutions. Informed consent for each study participant was obtained. The study was conducted in accordance with the European Good Clinical Practice requirements (Declaration of Helsinki).

### 2.3 Multiparameter flow cytometry

Cryopreserved PBMCs were thawed in 10 mL of RPMI 1640 (Gibco, Thermo Fisher Scientific) supplemented with 10% FBS and stained immediately without a resting period to optimize cell recovery. Cells were resuspended in Fixable Violet Viability kit (Thermo Fisher Scientific) following manufacturer´s protocol, incubated for 30 minutes at 4°C in the dark and washed twice with phosphate buffered saline (PBS) 1X (Gibco, Thermo Fisher Scientific). For each antibody configuration, one million cells per panel were incubated with 1:100 dilution of Fc receptor blocking solution containing purified monoclonal anti-CD16 antibody (CB16) (eBioscience) in staining buffer (PBS 1X with 2% bovine serum albumin (Sigma-Aldrich) and 2 mM EDTA (Sigma-Aldrich)) for 15 minutes at 4°C. Next, cells were stained according to the corresponding antibody panels. The same flow cytometry panel configuration was used for cohorts 1 and 2. For cohort 3, the panel configuration was revised to incorporate updated targets of interest (**Supplementary Table 2**). The following fluorochrome-conjugated antibodies diluted in staining buffer: anti-human-CD3-FITC (UCHT1), -CD3-PerCP (SK7), -CD8-V500 (RPA-T8), -CD25-Alexa Fluor700 (M-A251), -CD28-PE-Cy5 (CD28.2), -CD45RA-FITC (HI100), -CD57-APC (NK-1), -CD69-PE-Cy5 (FN50), -CCR7-PE (150503), -CXCR5-BB515 (RF8B2), and -HLA-DR-PE-Cy5 (G46-6) (BD Becton Dickinson, Franklin Lakes, NJ, USA); anti-human-NKG2A-PE (Z199) (Beckman Coulter); anti-human-CD3-APC-Cy7 (UCHT1), -CD3-PE (UCHT1), -CD4-PerCP-Cy5.5 (OKT4), -CD8-V500 (SK1), -CD11b-Alexa Fluor700 (ICRF44), -CD14-Brilliant Violet605 (G3D3), -CD14-Pacific Blue (M5E2), -CD14-PerCP-Cy5.5 (M5E2), -CD15-PE-Cy7 (W6D3), -CD19-PerCP (4G7), -CD27-Brilliant Violet605 (323), -CD39-APC (A1), -CD45-Brilliant Violet605 (HI30), -CD45RA-Alexa Fluor700 (HI100), -CD57-FITC (HNK-1), -CD73-PE-Dazzle594 (AD2), -CD85j-PE-Cy7 (GHI/75), -CD103-Brilliant Violet605 (Ver-ACT8), -CD137-PE-Dazzle594 (4B4-1), -CCR7-Alexa Fluor700 (G043H7), -CTLA-4-PE-Dazzle594 (BN13), -HLA-DR-PE-Cy7 (L243), -ICOS-APC-Cy7 (C398.4A), -KLRG1-PE-Dazzle594 (2F1), -LAG3-Brilliant Violet605 (1103065), -NKG2D-PE-Cy7 (1D11), -PD1-APC (EH12.2H7), -PD1-APC-Cy7 (EH12.2H7), -TCR-γ/δ-PE-Dazzle594 (B1), and -TIM3-Brilliant Violet605 (F38-2E2) (BioLegend); anti-human-CD16-APC-eFluor780 (CB16), -CD56-APC (CMSSB), -CD56-PE (CMSSB), -CD103-APC (B-Ly7), -TIM3-PE-Cy7 (F38-2E2) (eBioscience), and anti-human-NKG2C-Alexa Fluor700 (134591) (R&D Systems) for 30 minutes at 4°C in the dark. For intracellular markers, surface-stained cells were fixed and permeabilized following the protocol from BD Cytofix/Cytoperm Fixation/Permeabilization Solution Kit (BD Becton Dickinson) and incubated with the following antibodies: anti-human-Ki67-Alexa Fluor700 (B56) (BD Becton Dickinson); anti-human-FOXP3-PE (150D/E4), -Ki67-PE-Cy7 (20Raj1) (eBioscience); and anti-human-A2aR-PE (7F6-G5-A2) (Novus Biologicals) for 30 min at 4°C in the dark and finally washed twice and resuspended in 100 µL of PBS 1X until acquisition using a LSR Fortessa flow cytometer (BD Becton Dickinson). Instrument quality control was performed regularly by the CRG Flow Cytometry Core Facility. Healthy donor PBMCs and single-color controls were used for compensation. Gating thresholds for marker positivity were established using bimodal distribution for primary antigens (clear positive/negative separation) and Fluorescence Minus One (FMO) or isotype controls were used to define positivity thresholds for secondary (variable expression) and tertiary antigens (poorly resolved from negative populations). Samples from different time points were processed separately due to treatment effects on cell properties, with cytometer settings and compensation adjusted accordingly. Quality control was integrated into the flow cytometry acquisition phase; initial gating included debris removal (FSC vs. SSC) and doublet exclusion (FSC-A vs. FSC-H). To account for variable post-thaw viability (typically >75%), a LIVE/DEAD fixable dead cell stain was utilized in all panels. Only viable, singlet, non-debris events were included in the final biomarker analyses to ensure data stringency across all patient samples. Data obtained were analyzed using FlowJo software (v10.8.1) (BD Becton Dickinson) and a standardized gating strategy was applied to all samples within each longitudinal time point, to ensure analytical consistency and minimize operator-dependent bias. The accuracy of the gating strategy was further validated through back-gating of identified sub-populations to confirm their expected scatter and phenotypic characteristics. A sequential lineage-based gating strategy identified T cells, NK cells and MDSCs shown in **Supplementary Figure 1**, followed by subpopulation analysis within T and NK compartments. No significant associations were observed for MDSCs. Complete material references are listed in **Supplementary Table 3**.

### 2.4 Statistical analysis

We included 3 cohorts with different treatment strategies. Chemotherapy alone (cohort 1), chemotherapy plus ipilimumab (cohort 2) and chemotherapy plus anti PD-1/PD-L1 agents (cohort 3). Cohort 1 was used as a control for events specifically associated to outcome or toxicity from the ICBt in the other cohorts. PFS was calculated from the first cycle of ICBt until disease progression or death. OS was calculated from SCLC diagnosis until death. PFS and OS were defined according to standard criteria. In this clinical setting, multiple studies have demonstrated that OS represents a more robust and reliable endpoint. Accordingly, OS was selected as the primary outcome for association analyses in our study. All patients were included for PFS analysis, whereas one patient from cohort 3 was excluded from OS analysis due to diagnosis at stage III and late relapse at which point samples for the current study were obtained.

OS and PFS comparisons between groups were performed using Kaplan-Meier survival curves and the log-rank test. To detect the best cutoff of time-to-event–prediction variables, the maximally selected rank statistics (Maxstat) method was used.

Dimensionality reduction was performed using Principal Component Analysis (PCA), which represents high-dimensional data by fewer principal components without losing major information (21). For the different markers assessed in the 3 cohorts, PCA and contribution assessment were performed using the *prcomp* function from the R stats package.

For categorical data, comparisons of proportions were evaluated by χ2 test or Fisher’s exact test, when appropriate. For continuous variables, comparisons were assessed by nonparametric Mann-Whitney U or Kruskal-Wallis test, when appropriate. Spearman rank correlation was used to evaluate the strength of association between 2 continuous variables.

Normalized expression of the different markers was calculated using the formula:


$$\frac{\left(TP2i-TP1i\right)}{TP1i}\;\times 100$$

TP1 and TP2 represent the baseline value and posttreatment value, respectively. The cutpoints chosen for each analysis are shown in the **Supplementary Table 4**.

Data analysis was performed with R software version 4.1.2 and P values ≤0.01 and <0.05 were set as the significance level for cellular subsets analysis and survival analysis, respectively.

Results from overall correlative analyses should be considered descriptive because of the small number of samples and unadjusted sequential testing. P values and 95% CI do not represent a confirmatory measure of clinical or statistical relevance, but a rough reference for designing future, hypothesis-driven studies and informing investigators about the uncertainty of our exploratory data.

## 3 Results

### 3.1 Study group population

The majority of patients were male in the 3 cohorts (70.5, 64.9, and 80% in cohorts 1, 2, and 3, respectively), with age range means from 62.6 to 67.6 years old. Most of the patients presented with a performance status 1 (58.3, 54.1, a 45% respectively), stage IV of the disease at diagnosis (100% in cohorts 1 and 2, and 80% in cohort 3) and were either active or former smokers (**Table 1**). Overall, patient characteristics were broadly comparable across the three cohorts in terms of age, sex, and smoking status. Cohort 2 included patients from a different geographical region. Although these samples are highly valuable, their limited availability and the inherent heterogeneity of the patient population may have contributed to the observed results.

**Table 1:** Baseline clinical characteristics of SCLC patients included into 3 cohorts.

Clinical characteristic	Cohort 1	Cohort 2	Cohort 3	
	Chemotherapy alone	Chemotherapy + anti-CTLA-4	Chemotherapy + anti-PD-1/PD-L1	*P value*
	N=24	N = 37	N = 20	
Gender, N (%)				*ns*
Female	7 (29.5)	13 (35.1)	4 (20)	
Male	17 (70.5)	24 (64.9)	16 (80)	
Age				*ns*
Mean	67,6	62,6	66,5	
Range	52 – 83	44 – 84	55 - 84	
Performance status, N (%)				*ns*
0	4 (16.6)	11 (29.7)	8 (40)	
1	14 (58.3)	20 (54.1)	9 (45)	
2	4 (16.6)	None	1 (5)	
3	2 (8.3)	None	1 (5)	
NA	None	6 (16.2)	1 (5)	
Stage, N (%)				*p=0.006*
I-III	None	None	4 (20)	
IV	16 (100)	37 (100)	16 (80)	
Smoking status, N (%)				*ns*
Smoking history	24 (100)	10 (27.1)	20 (100)	
Never smokers	None	None	None	
NA	None	27 (72.9)	None	
Immune-related adverse events, N (%)				
Grade 0 - 2	–	11 (30)	19 (95)	
Grade 3 - 4	–	26 (70)	1 (5)	
Progression-free survival, months				
Median	4,9	6,93	6,32	*p=0.007*
95% CI	4.2-6.3	5.91-8.44	4.83-20.23	
Overall survival, months				
Median	8,3	17,01	8,82	*ns*
95% CI	7.2-13.3	8.8-NA	6.7-26.5	

NA, data not available; ns, no significance.

**Figure 1:**
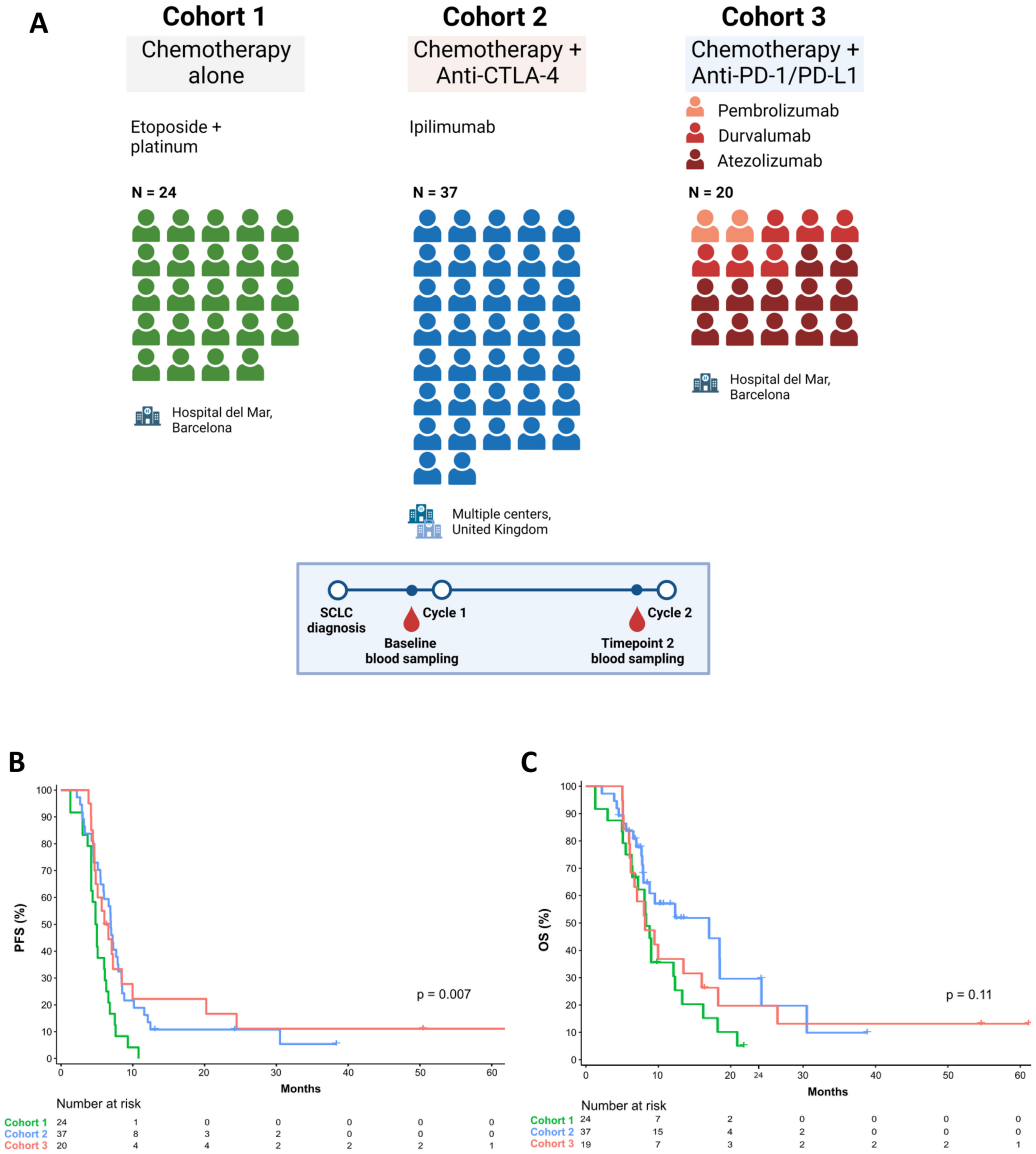
Patients’ cohorts for PBMC analyses and clinical outcomes. **(A)** PBMCs were collected from 3 different patient cohorts: Cohort 1: treated with chemotherapy (etoposide + platinum, N = 24); Cohort 2: chemotherapy + anti-CTLA-4 (ipilimumab, N = 37) (ICE-trial); Cohort 3: chemotherapy + anti-PD-1/PD-L1 (pembrolizumab, durvalumab, or atezolizumab, N = 20). Samples were collected before treatment initiation and at Cycle 2. Illustration created with BioRender.com. **(B)** Kaplan-Meier curves for PFS and **(C)** OS.

All 24 patients from cohort 1 were treated exclusively with platinum plus etoposide-based chemotherapy and did not receive immunotherapy in subsequent lines, while the 37 patients from cohort 2 were additionally treated with the anti-CTLA-4 agent ipilimumab (a fully human IgG1κ) at 10mg/kg. From the total 20 patients included into cohort 3, 2 patients received pembrolizumab (an anti-PD-1 humanized IgG4κ) at 200 mg, 6 were treated with durvalumab (an anti-PD-L1 fully human IgG1κ) at 1500 mg, and 12 patients received atezolizumab (an anti-PD-L1 humanized IgG1) at 1200 mg in addition to chemotherapy (**Figure 1A**).

Survival analysis comparing all 3 cohorts showed a significant 2-months improvement in PFS in those patients treated with either anti-CTLA-4 or anti-PD-1/PD-L1 immunotherapy with medians of 6.93 and 6.32 months in cohorts 2 and 3 respectively, when compared to the 4.9 months PFS in the chemotherapy alone cohort (p=0.007*)* (**Figure 1B**). However, there was no statistically significant difference in 2-years OS (**Figure 1C**).

### 3.2 Baseline immune cell subpopulations were associated with outcomes and toxicity after treatment with anti-CTLA-4 therapy

**Figure 2:**
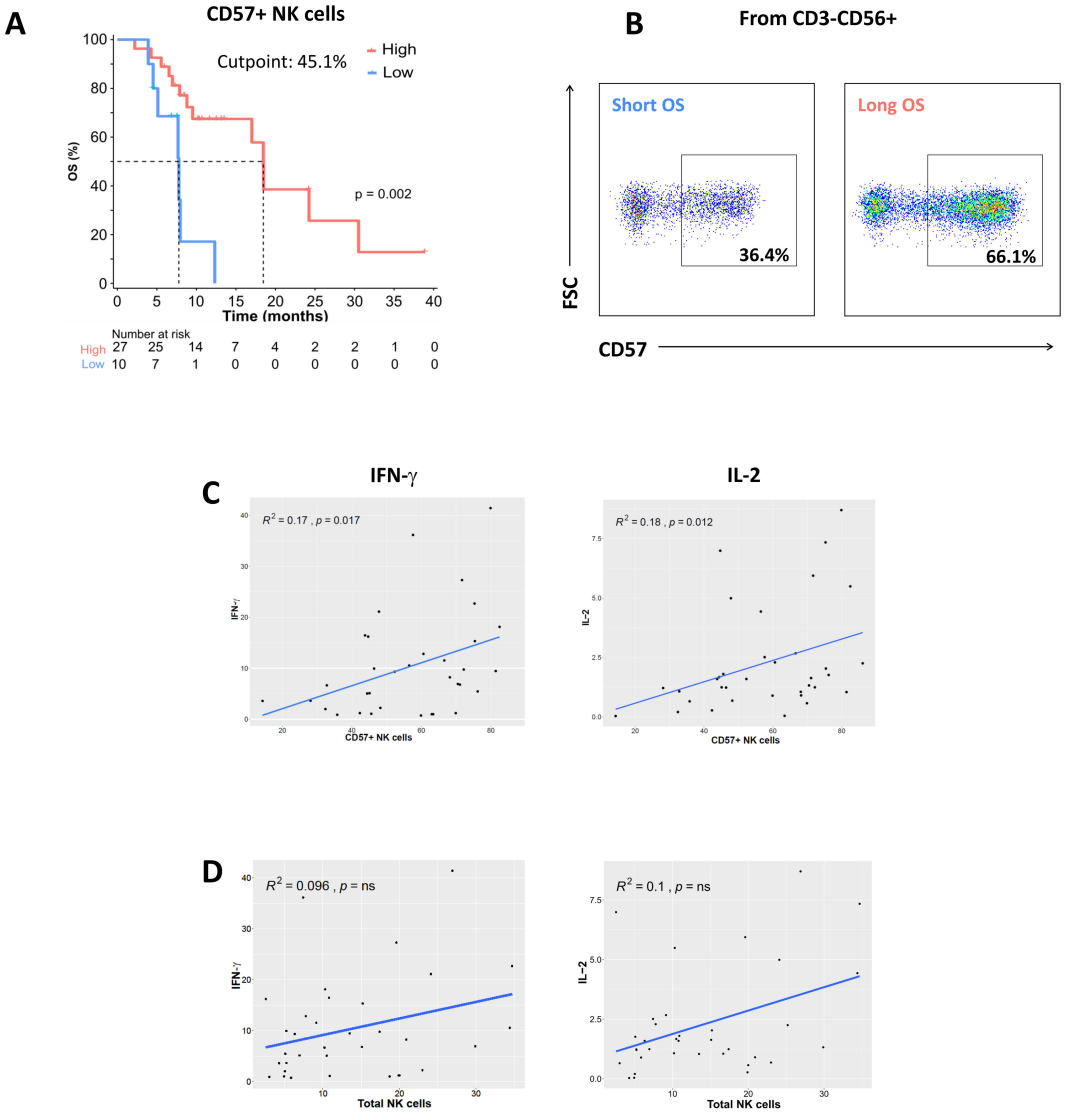
Baseline immune populations and cytokines and their association with outcomes in patients. **(A)** Kaplan–Meier survival curves for patients with SCLC stratified by CD57^+^ NK cell percentage in cohort 2 (ipilimumab + chemotherapy). **(B)** Representative flow cytometry plots of CD57 expression on CD3^-^CD56^+^ NK cells in patients with short versus long OS. **(C)** Spearman correlation between CD57^+^ NK cell percentage and IFN-γ and IL-2 production. **(D)** Spearman correlation between total NK cells and IFN-γ and IL-2 production.

To identify those cellular subsets with the greatest contribution for the differentiation of each cohort, a principal component analysis (PCA) was conducted. Then, identified cell subpopulations were tested against OS. We found that in NK cells, expression of the differentiation and cytotoxicity marker CD57 above the calculated cutpoint of 45.1% could efficiently discriminate those patients with longer OS in cohort 2 (**Figures 2A, B**). High expression of CD57 in NK cells was a predictor of benefit from anti-CTLA-4 therapy in a specific manner, as this association was not found in patients treated with chemotherapy alone (cohort 1) or with anti-PD-1/PD-L1 blockade (cohort 3) (**Supplementary Figure 2**).

We previously reported that the anti-CTLA-4 agent ipilimumab induces a global increase in Th1, Th2, and inflammatory cytokines in SCLC patients from cohort 2 (20). The serum cytokine quantification data used in the present study were entirely derived from this previously published work; no additional cytokine measurements were performed. These cytokine data were integrated with the newly generated PBMC immunophenotyping data to explore immune correlates of clinical outcome. Specifically, we investigated whether patients with longer OS and higher proportions of CD57+ NK cells showed an antitumoral cytokine profile. Spearman correlation analysis of the previously reported cytokine measurements in patients from cohort 2 revealed a linear correlation between expression of CD57+ NK cells and serum Th1 cytokines IFN-γ (r^2^ = 0.17, p=0.017) and IL-2 (r^2^ = 0.18, p=0.012) (**Figure 2C**). This correlation was not observed with total NK cells or other cytotoxic and effector T cell populations (2D, **Supplementary Figure 3**). Although modest, the associations are biologically plausible because CD57^+^ NK cells represent a mature NK-cell subset with established functional links to cytokine responsiveness and IFN-γ production (22).

### 3.3 T cell subsets identify long survival patients treated with PD-1/PD-L1 axis blockade

**Figure 3:**
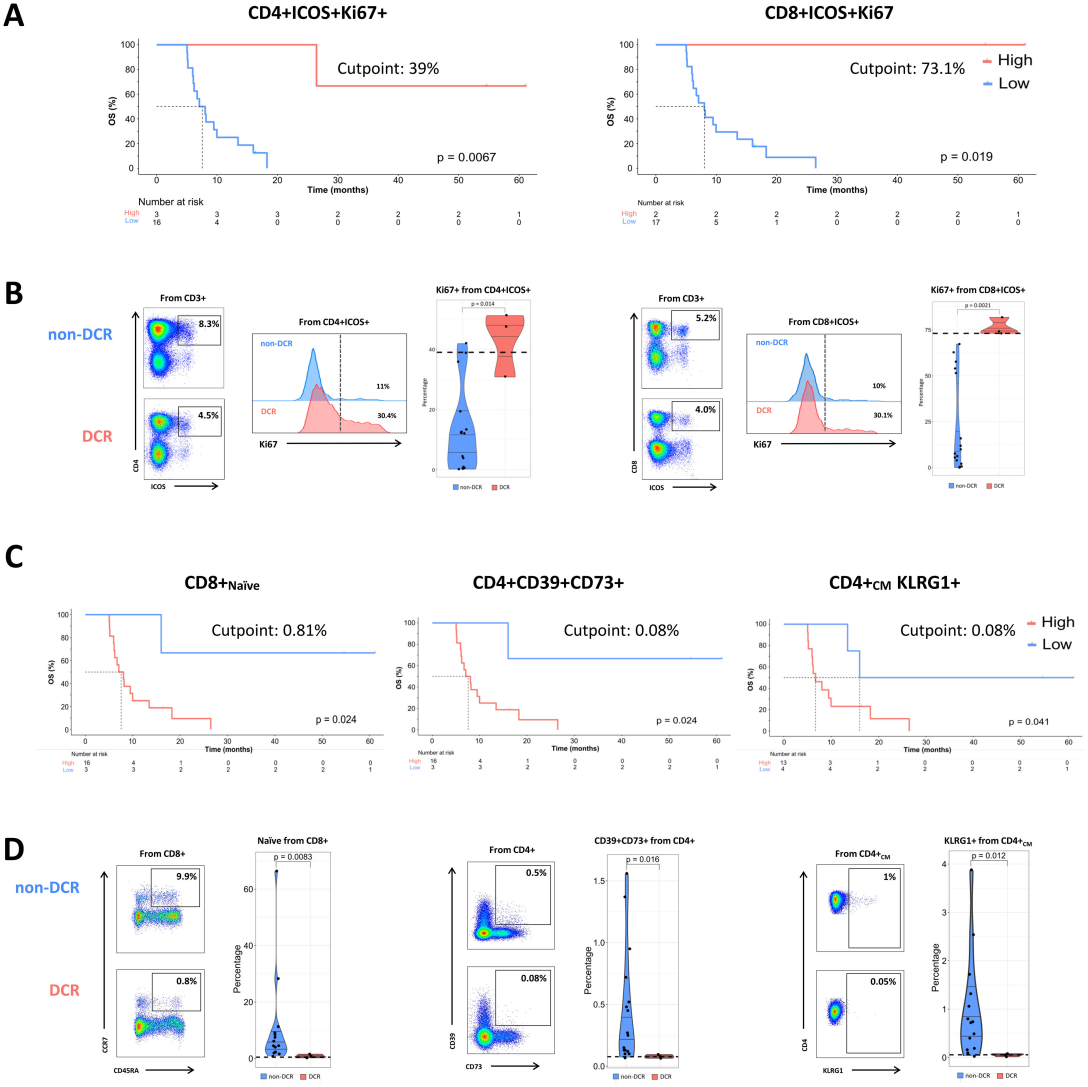
Baseline T-cell subsets and their association with benefit from treatment. **(A)** Kaplan–Meier curves for OS of patients from cohort 3 (chemotherapy + anti-PD-1/L-1) stratified by baseline CD4^+^ICOS^+^Ki67^+^ and CD8^+^ICOS^+^Ki67^+^ T cells. **(B)** Flow cytometry plots showing Ki67^+^ frequencies within CD4^+^ICOS^+^ and CD8^+^ICOS^+^ populations in DCR vs. non-DCR patients in cohort 3. **(C)** Kaplan–Meier curves for OS in DCR vs. non-DCR patients in cohort 3 stratified by CD8^+^ naïve, CD4^+^CD39^+^CD73^+^, and CD4^+^CM KLRG1^+^ subsets. **(D)** Flow cytometry plots comparing CD8^+^ naïve, CD4^+^CD39^+^CD73^+^, and CD4^+^CM KLRG1^+^ frequencies in DCR vs. non-DCR patients. DCR, durable clinical response. Cutpoints were defined by maximally selected rank statistics (Maxstat) to maximize discrimination of OS.

Upregulation of the proliferation marker Ki67 in peripheral T cells in response to ICBt has been previously reported (23–25). Accordingly, we analyzed the expression of Ki67 in several T cell subsets from patients from our three cohorts (**Supplementary Tables 5**–**7**). In cohort 3, the percentages of Ki67-expression along with the costimulatory receptor ICOS in CD4+ and CD8+ T cells were significantly associated with improved OS. Baseline high proportions of Ki67 in CD4+ICOS+ (above 39%) and CD8+ ICOS+ T cells (above 73.1%) showed a correlation with improved outcome (p=0.0067 and 0.019, respectively) (**Figure 3A**). Interestingly, preexisting CD8+ICOS+Ki67+ T cells also showed an impact in survival in patients treated with chemotherapy alone (cohort 1; p=0.03) (**Supplementary Figure 4**) suggesting that the presence of this cell subset may be an overall good prognostic marker rather than predicting benefit specifically from anti-PD-1/PD-L1 blockade.

In an additional approach, we studied the predictive power of these immune subsets for duration of response. To dichotomize patients from cohort 3 into those exhibiting a durable clinical response (DCR) and those without a durable clinical response (non-DCR), we arbitrarily set the cutpoint in 200 days based in our PFS data distribution. As expected, only 3 (15%) patients met the criteria to be included in the DCR group while the majority of patients were classified into the non-DCR group (N = 17, 85%). We found that those patients in the DCR group consistently showed baseline increased proportions of Ki67 expression in CD4+ICOS+ and CD8+ICOS+ T cells (P = 0.012; P = 0.0018 respectively) when compared to the non-DCR group (**Figure 3B**).

Notably, we also noted baseline specific cellular subsets associated to poor survival. We observed that pretreatment high percentages of CD8+_Naïve_ T cells (p=0.024), CD39+CD73+ double positive CD4+ T cells (p=0.024), and KLRG1+ in the central memory subset of CD4+ T cells (CD4+_CM_ T cells) (p=0.041) were present in those patients with worse OS (**Figure 3C**). In a tumor setting, these 3 cell subsets may be related to unresponsiveness to antigen stimulation (26), adenosine-mediated immune suppression (27), and T cell exhaustion (28), respectively. Of note, calculated cutpoints for each one of these populations were markedly low (0.81% for CD8+_Naïve_, 0.08% for CD4+CD39+CD73+, and 0.08% for CD4+CM KLRG1+ T cells). When we further analyzed these immune phenotypes in terms of DCR, we observed that all 3 patients from the DCR group showed almost null percentages of these cell subsets (p=0.0083, p=0.016, and p=0.012 for CD8+_Naïve_, CD4+CD39+CD73+, and CD4+_CM_ KLRG1+ T cells respectively) (**Figure 3D**). Given the small sample size, these observations remain descriptive and require validation in larger cohorts.

### 3.4 Modulation of proliferation and antigen-sensitization markers following ICBt is associated with better prognosis

**Figure 4:**
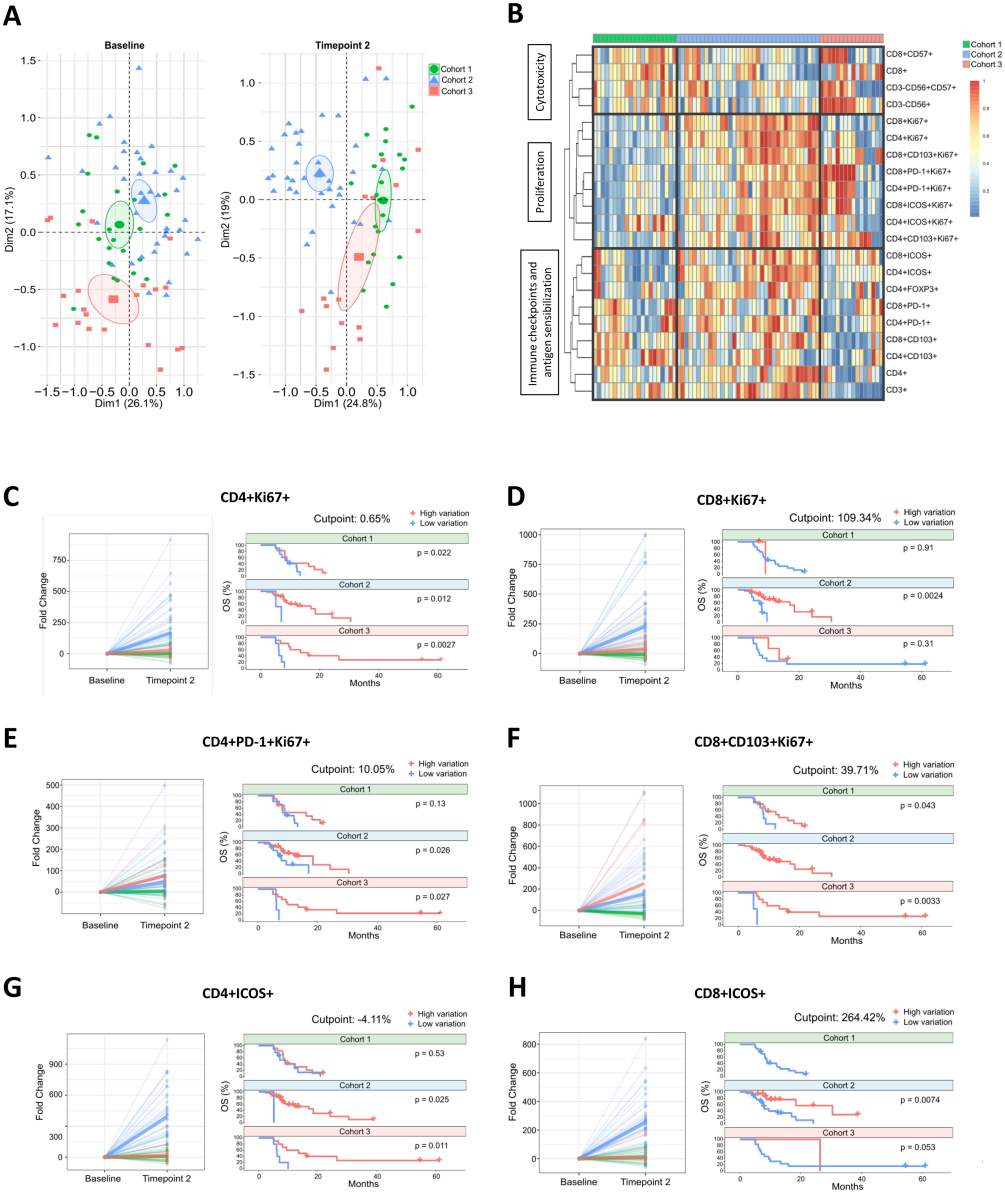
Longitudinal changes in immune cell subsets and survival associations. **(A)** Principal component analysis plots at baseline and timepoint 2 showing patient clustering by immune cell profiles across three cohorts. **(B)** Normalized variation heatmap showing clustering of immune cell subsets based on cytotoxicity, proliferation and immune checkpoint and antigen sensibilization for each cohort. **(C–H)** Waterfall plots depicting longitudinal changes (baseline to timepoint 2) and Kaplan–Meier curves showing survival associations (high vs. low variation) for: **(C)** CD4^+^Ki67^+^, **(D)** CD8^+^Ki67^+^, **(E)** CD4^+^PD-1^+^Ki67^+^, **(F)** CD8^+^CD103^+^Ki67^+^, **(G)** CD4^+^ICOS^+^, and **(H)** CD8^+^ICOS^+^. Cutpoints were defined by maximally selected rank statistics (Maxstat) to maximize discrimination of OS.

We evaluated the immune kinetics triggered in response to therapy of 22 shared immune subsets among the 3 cohorts (**Supplementary Table 8**). A PCA was performed for baseline and post-treatment (Timepoint 2) pooled data from all 3 cohorts. We observed that before treatment there was no substantial clustering among cohorts, however, after initiation of treatment each cohort showed a tendency to cluster. Interestingly, patients from cohort 2 showed a more recognizable clustering than patients that received chemotherapy alone or anti-PD-1/PD-L1 therapy, suggesting a more pronounced immune modulation mediated by CTLA-4 blockade (**Figure 4A**) probably related to the earlier impact of CTLA-4 modulation in the immune cycle and therefore a broader and more nonspecific immune activation (29). The normalized variation heatmap showed clustering of immune subsets based in cytotoxicity, proliferation, and immune checkpoints and antigen sensibilization in a cohort-dependent manner (**Figure 4B**).

We identified 6 main cellular subsets which modulation after the initiation of ICBt correlated with survival. First, we observed that anti-CTLA-4 therapy induced an upregulation of Ki67 in a more profound manner than the anti-PD-1/PD-L1 therapy (**Figures 4C–F**). In fact, a slight increase of 0.65% in the baseline proportion of total CD4+Ki67+ T cells was able to identify patients with improved OS in both ICBt cohorts 2 and 3 (p=0.012 and 0.0027, respectively) (**Figure 4C**). A more robust variation in the expression of Ki67 was required in the CD8+ T cell subset (109.34% relative increase from baseline) to efficiently identify long survivors and was restricted to patients treated with anti-CTLA-4 therapy (p=0.0024) (**Figure 4D**).

An increase in Ki67 expression in CD4+PD-1+ T cells above a cutpoint of 10.05% relative to baseline was associated with improved OS in cohorts 2 (p=0.026) and 3 (p=0.027) (**Figure 4E**). In addition, an expansion of Ki67-expressing CD8+CD103+ T cells of more than 39.71% relative to baseline identified patients with increased survival in cohorts 1 and 3 (p=0.043 and 0.0033, respectively) (**Figure 4F**). Interestingly, anti-CTLA-4 therapy induced an upregulation of this immune cell subset in all of the patients studied in cohort 2.

Notably, we also observed that a negative modulation of ICOS in CD4+ T cells of -4.11% from baseline allowed to identify those patients with shorter survival in both anti-CTLA-4 and anti-PD-1/PD-L1 cohorts (p=0.025 and 0.011, respectively) (**Figure 4G**). On the contrary, an upregulation of ICOS in total CD8+ T cells above a cutpoint of 264.42% relative to baseline identified long survivors only in cohort 2 (p=0.0074) (**Figure 4H**).

### 3.5 CD137-expressing CD4+T cells predispose to immune-related colitis in patients treated with anti-CTLA-4 therapy

**Figure 5:**
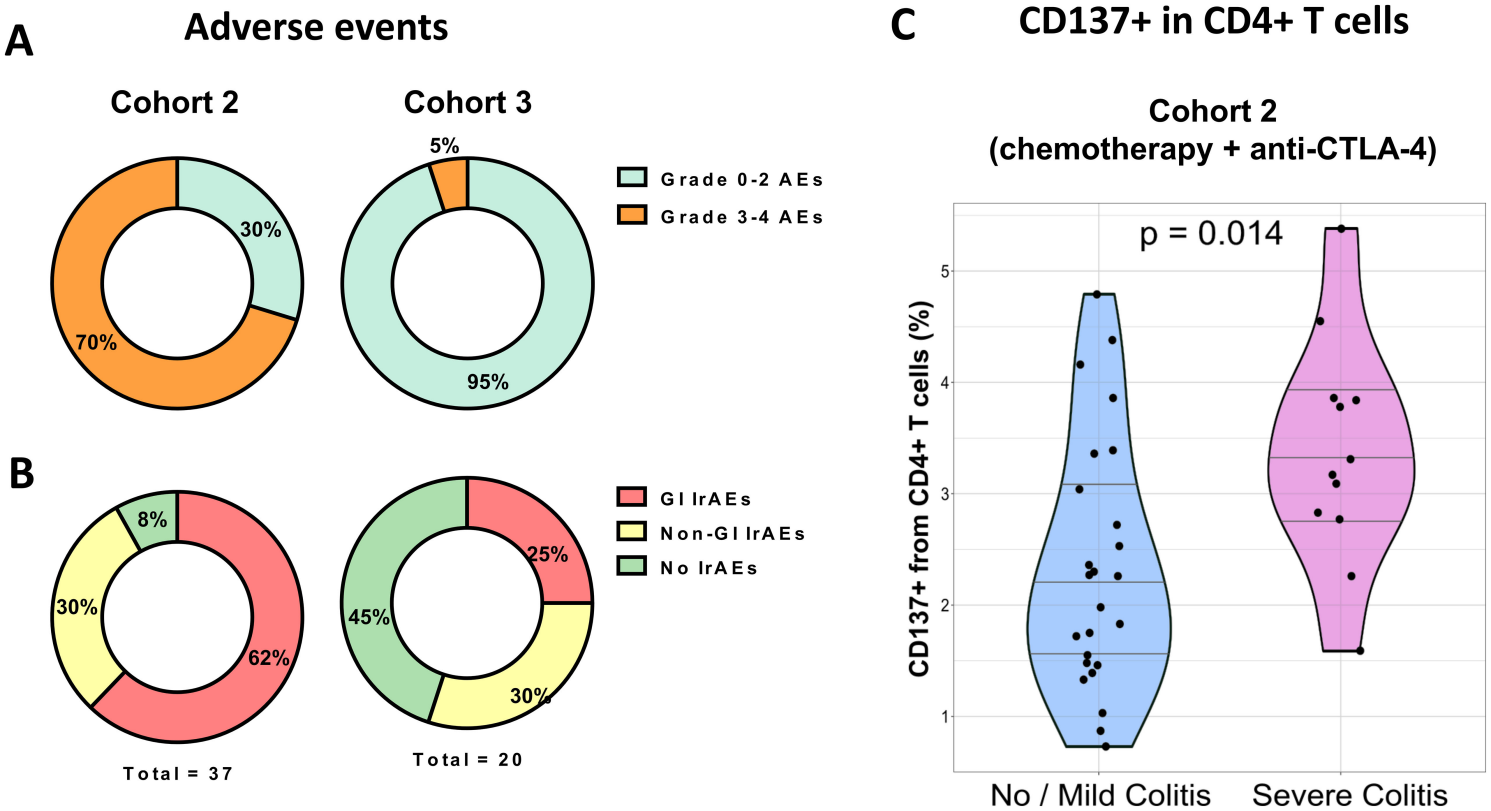
Adverse events and CD137 expression in CD4^+^ T cells. **(A)** Distribution of immune-related adverse event (irAE) severity (Grade 0–2 vs. 3–4) in Cohort 2 (chemotherapy + anti–CTLA-4) and Cohort 3 (chemotherapy + anti–PD-1/PD-L1). **(B)** Distribution of irAE types (GI, non-GI, or none) in cohort 2 and cohort 3. GI, gastrointestinal. **(C)** Violin plot of CD137^+^ frequency in CD4^+^ T cells comparing no/mild vs. severe colitis in Cohort 2.

Checkpoint inhibitors are well known for the development of a unique profile of immune-related adverse events (irAEs) (30). Accordingly, we aimed to investigate any association between the distribution of cell subsets and irAEs in patients treated with ICBt. Toxicity was of lower grade and less frequent in patients treated with anti-PD-1/PD-L1 agents (**Figure 5A**) and no associations were found with peripheral immune subpopulations (data not shown). Of note, 23 out of the 37 patients (62%) treated with chemotherapy + ipilimumab developed any grade of gastrointestinal irAEs (**Figure 5B**). Characterization of circulating immune cells showed that those patients who suffered a severe grade 3 or 4 colitis from cohort 2 (12 patients, 32.4%) expressed higher basal proportions of the activation marker CD137 in CD4+ T cells (p=0.014), in contrast with those patients who never developed or presented mild (grade 1 or 2) ipilimumab-associated colitis (**Figure 5C**).

## 4 Discussion

Exploitation of the anti-tumor immune response by ICBt has irrupted as a hopeful therapeutic strategy in SCLC despite modest survival benefit (31–33). Due to the low proportion of long-term survivors, the effectiveness of ICBt in front-line therapy of unselected SCLC patients is being questioned (34). In addition, new therapeutic strategies (i.e. DLL3 targeting agents) are being tested in the first line setting in combination with standard chemoimmunotherapy with promising results but added toxicity (NCT06077500, NCT05361395). In this potential future scenario, being able to identify those patients that benefit from each strategy (anti-PD-1/PD-L1 agent or T-cell engagers) is going to be crucial to save in costs (if either drug is not needed) and toxicity of unnecessary agents. Therefore, there is an urgent need for predictive biomarkers of response for an optimal selection of patients for each treatment. Here, we identified pretreatment immune subsets and ICBt-driven immune kinetics in peripheral blood correlated with prediction of benefit from anti-CTLA-4 or anti-PD-1/PD-L1 agents in combination with chemotherapy in patients with ED-SCLC.

When treated with chemotherapy plus anti-CTLA-4 agent ipilimumab, our results showed that pretreatment CD57+ NK cells above cutpoint correlated with better survival. In fact, CD57 is a terminal-differentiation marker that induces enhanced cytotoxicity and intrinsic production of IFN-γ in NK cells (22). In line with this, we previously reported that SCLC patients who benefit from anti-CTLA-4 therapy showed higher baseline levels of IL-2 (20). Our analysis revealed a positive correlation of CD57-expressing NK cells with inflammatory cytokines IL-2 and IFN-γ levels, suggesting that patients with baseline high proportions of CD57+ NK cells in peripheral blood display a systemic proinflammatory status which ultimately may contribute to an improved anti-tumor response and survival. Nevertheless, the prognostic impact of circulating CD57^+^ NK cells appears to be tumor- and treatment-dependent, as high frequencies of circulating CD57^+^ NK-cell have been associated with resistance to HER2 antibodies in breast cancer (35).

Moreover, pretreatment high proportions of Ki67 in CD4+ICOS+, and CD8+ICOS+ T cells predicted long survival in patients treated with chemotherapy plus anti-PD-1/PD-L1 therapy. Ki67 is expressed in all phases of the cell cycle except for G0, and its expression in specific immune subsets may differentiate quiescent from proliferative activated cells (36). ICOS is a receptor expressed on activated and memory T cells. ICOS interaction with its ligand B7RP-1 promotes T-cell proliferation and cytokine production (37). However, the role of ICOS is still not well-defined and has differed over the years (38, 39). In fact, both ICOS antagonist and agonist approaches have been proposed in cancer therapy (40). Our results highlight the role of Ki67 in these specific immune subsets, since predictive properties of CD4+ICOS+ and CD8+ICOS+ T cell subsets were lost when Ki67 was not considered for analysis (data not shown).

In our analysis we also evaluated associations between baseline immune profiles and durable clinical response (DCR). As anticipated, only a small number of patients met the criteria, reflecting the aggressive nature of this disease. We acknowledge that these analyses are limited by small sample size and the use of a data-driven cutoff and should therefore be considered hypothesis-generating.

We report that high basal percentages of CD4+CD39+CD73+, CD8+_Naïve_, and CD4+_CM_ KLRG1+ T cells correlated with shorter OS in patients treated with anti-PD-1/PD-L1 agents. Indeed, when CD39 and CD73 are co-expressed, they dephosphorylate extracellular ATP into adenosine inducing direct immunosuppressor effects and blocking the differentiation of naïve into effector T cells (41), while the co-inhibitory receptor KLRG1 has been classically considered as a senescence marker (28, 42). Of note, cutpoints for these 3 cell populations were markedly low, suggesting that the sole presence of these dysfunctional subsets may reflect a profound basal impaired immune response against SCLC that cannot be reverted with anti-PD-1/PD-L1 blockade. As these specific populations were analyzed only in the cohort 3, one limitation of our study is that we do not know whether these immune populations might be related with poor prognosis independently of the ICBt strategy. Another group has reported that patients with ES-SCLC treated with chemoimmunotherapy with high CD3+CD8+PD-1+ T-cell levels together with PD-L1+ CTCs at baseline show better survival (43). A recent paper has evaluated biomarkers of benefit from immunotherapy in these patients and has reported that higher levels of monocyte-dendritic cells in pre-treatment PBMCs were found in durable clinical benefit group (44). However, in these studies there was no control group treated with chemotherapy alone, precluding the possibility of distinguishing between the prognostic or predictive role of this subpopulation. Despite the use of different agents targeting the PD-1/PD-L1 axis, current evidence from several meta-analyses indicates no significant differences in efficacy, as measured by PFS or OS, among these therapies (45). Furthermore, a focused review of peripheral blood immune cell biomarkers in the context of anti–PD-1/PD-L1 treatment has integrated findings from studies involving nivolumab, pembrolizumab, atezolizumab, and durvalumab. This review highlights consistent immunological patterns, such as the expansion of activated or PD-1^+^ CD8^+^ T cells, modulation of myeloid cell subsets, and remodeling of the T-cell receptor (TCR) repertoire, without systematically distinguishing between PD-1 and PD-L1 inhibitors (46). These observations support the interpretation of our results across different anti–PD-1/PD-L1 agents and suggest that the immune dynamics identified here may reflect shared mechanisms of response to checkpoint blockade rather than drug-specific effects.

The immune modulation of circulating immune cells after ICBt has been scarcely explored in SCLC. One recent report, observed that CD8+ cytotoxic T-cell frequencies decline was associated with early relapse risk (47). Other studies have analyzed changes in peripheral T cell receptor (TCR) repertoire in SCLC (48, 49) and exhaustive characterization of circulating immune subsets in cancer patients along anti-PD-L1 therapy were not capable to identify any significant immune modulation (16).

Remarkably, Ki67 was not considered for such studies. In this work, Ki67 has been revealed as a key marker for OS prediction, where an increase in proportions of proliferative Ki67-expressing CD4+ and CD8+, CD8+CD103+, and CD4+PD-1+ T cells correlated with improved survival in patients after treatment with either of the two ICBt strategies. Our findings are consistent with studies in other tumor types showing that early expansion of Ki67+CD8+ T cells is associated with clinical benefit in NSCLC and melanoma patients treated with ICBt (24, 25). Together, these data support the concept that treatment-induced proliferation of peripheral T cells may serve as an early dynamic biomarker of response. In line with this, CD103+ T cells have been proposed as a potential biomarker of response to PD-1/PD-L1 blockade across multiple tumor types. Intratumoral CD103+CD8+ T cells have been shown to predict response to either PD-1 therapy in NSCLC (50) or PD-L1 therapy in NSCLC and metastatic urothelial carcinoma (51). Notably, these observations have been largely derived from analyses performed on paraffin-embedded tumor tissue samples, reflecting immune dynamics within the tumor microenvironment. Similarly, analyses performed on PBMC samples of NSCLC and gastric cancer patients treated with anti-PD-1/PD-L1 therapy suggested that CD103 expression in circulating T cells may also have predictive value (52, 53). In this context, our findings extend these observations to SCLC and further indicate that dynamic changes in circulating CD103+ T-cell populations may capture clinically relevant antitumor immune responses in a minimally invasive manner, which is particularly relevant in a disease where tissue availability is limited. Of note, the expansion of tissue-resident CD8+CD103+ T cells served as an OS predictor also in patients treated with chemotherapy alone, suggesting that this population might identify patients with improved prognosis independently of treatment regimen. Interestingly, a downregulation of CD4+ICOS+ T cells in patients treated with either anti-CTLA-4 or anti-PD-1/PD-L1 was associated to better survival. We speculate that this association may be due to decrease of ICOS-expressing immunosuppressive cells, or relocation of tumor-reactive CD4+ICOS+ T cells into secondary/tertiary lymphoid organs and tumor bed.

As ICBt exploits the upregulation of immune activation, immune-mediated AEs are common (54). Predisposition to gastrointestinal IrAEs in anti-CTLA-4 therapy may be related to immune events as imbalance in microbiota (55), infiltration of CD4+ T cells and increased CD8+/FoxP3+ ratio (19, 56). An increased incidence of severe grade 3 or 4 IrAEs was observed in patients treated with anti-CTLA-4 therapy, consistent with reports showing higher-grade toxicity with anti-CTLA-4 compared other ICBt (57). Our results showed that high proportions of CD137-expressing CD4+ T cells can predict severity of colitis in patients treated with anti-CTLA-4 therapy. In fact, CD137 has been described on T cells of inflamed gut (58, 59), and our results may support the evaluation of CD137 in peripheral CD4+ T cells for assessment of predisposition to severe colitis in SCLC patients when anti-CTLA-4 therapy is indicated.

Whether any of our findings in peripheral blood reflect those immune events occurring within the tumor microenvironment is not known. From our understanding, findings in the periphery may directly or inversely reflect immune events occurring in the tumor bed, and might be affected by several factors as tumor heterogenicity (60), circulating tumor cells or metastasis (61). To respond these questions, further functional studies are needed to decipher the ICBt-driven immune events occurring in SCLC. Based in our results, characterization of peripheral immune subsets before the initiation of ICBt may help clinicians to recognize those patients who may benefit the most and to evaluate the risk for developing IrAEs, or even unfavorable survival prognosis.

In summary, we report hypothesis generating results describing peripheral immune subsets that predict prognosis to ICBt in SCLC. We show CD57 in NK cells and CD137 in T helper cells as predictive biomarkers for survival and IrAEs respectively for anti-CTLA-4 therapy, whereas Ki67 in peripheral CD4+ and CD8+ T cells that coexpress ICOS was unveiled as a key marker to identify long survivors in anti-PD-1/PD-L1 therapy. Conversely, baseline high proportions of CD8+_Naïve_, CD4+CD39+CD73+, and CD4+_CM_ KLRG1+ T cells are correlated to short survival. Finally, in both anti-CTLA-4 and anti-PD-1/PD-L1 treatments, upregulation of Ki67 in CD4+ and CD8+CD103+ T cells, as well as downregulation of CD4+ICOS+ T cells after ICBt were associated to improved prognosis.

We acknowledge the inherent heterogeneity across the three study cohorts, which differ in geographical origin, enrollment periods, and the specific assay panels utilized. These variations, arising from the use of a single-center Barcelona cohort (Cohort 1), a multicenter UK phase II trial (Cohort 2), and a later-recruited cohort from the same Barcelona institution (Cohort 3), may complicate the distinction between treatment-specific immune signatures and broader cohort-related effects. Correlations with baseline PBMC subpopulations might be affected by these differences; however, the longitudinal observations were normalized to each patient’s baseline, addressing potential cohort-associated bias. Furthermore, it is important to emphasize that the longitudinal biological samples analyzed in this study are exceptionally rare and difficult to obtain within these specific clinical contexts. While the differences in assay consistency between the original and revised panels may limit direct cross-cohort comparisons for rare cell populations, the observed trends provide a unique window into immune dynamics that would otherwise be inaccessible. Consequently, our findings should be interpreted as hypothesis-generating, serving as a biological foundation for future prospective studies using standardized platforms and more uniform patient populations.

In the context of limited available data, our findings add to emerging evidence suggesting that early on-treatment changes in peripheral immune subsets, interpreted relative to each patient’s own baseline rather than through single-timepoint inter-patient comparisons, may have predictive relevance for ICBt outcomes in SCLC. Although these observations are hypothesis-generating and require validation in larger cohorts, evaluation of the peripheral immune subsets reported here may help identify patients most likely to benefit and improve the cost-effectiveness of chemoimmunotherapy strategies in this setting.

## Data Availability

The original contributions presented in the study are included in the article/**Supplementary Material**. Further inquiries can be directed to the corresponding author.
