# Supplementary material for: Peripheral immune cell subsets as potential predictors of benefit from immune checkpoint blockade therapy in small cell lung cancer
Source: Front Immunol. 2026 Jun 12;17:1802274. doi: 10.3389/fimmu.2026.1802274 (PMC13303708; doi:10.3389/fimmu.2026.1802274)
Supplement: Supplementary file 5 [file Table1.docx]

**Supplemental Figure 1: Gating strategy for immune cell subsets**
Representative images of the flow cytometry gating strategy used to identify peripheral immune cells. T cells: Lymphocytes and singlets were gated, followed by CD3⁺CD19⁻ selection. CD4⁺ and CD8⁺ subsets were identified and further characterized by CCR7/CD45RA expression to define naïve, CM (central memory), EM (effector memory), and TEMRA (terminally differentiated effector memory RA) populations. NK cells: Lymphocytes and singlets were gated, then CD3⁻CD19⁻CD56⁺ cells were identified and stratified by CD16/CD56 expression. MDSCs (Myeloid-Derived Suppressor Cells): Live cells and singlets were gated, then CD3⁻CD19⁻HLA-DR⁻ cells were selected. Granulocytic MDSCs (G-MDSC: CD11b⁺CD15⁺CD14⁻) and monocytic MDSCs (M-MDSC: CD11b⁺CD14⁺CD15⁻) were identified within this population.

**Supplemental Figure 2: Overall survival (OS) in CD57⁺ NK cell subgroups**. Kaplan–Meier curves of OS in patients, stratified by CD57⁺ NK cell levels (high vs. low) across treatment cohorts. Top panel: Chemotherapy alone (Cohort 1); Bottom panel: Chemotherapy plus anti–PD-1/PD-L1 (Cohort 3).

**Supplemental Figure 3: Correlation of IFN-γ and IL-2 production with T-cell subsets**. Scatter plots showing correlations between cytokine production and T-cell subsets. Top rows: IFN-γ levels vs. CD4⁺ subsets (CD4pos_1, CD4_TEM, CD4_TEMRA) and CD8⁺ subsets (CD8pos_1, CD8_TEM, CD8_TEMRA). Bottom rows: IL-2 levels vs. the same T-cell subsets. R² and p-values indicate weak or non-significant correlations across all subsets. TEM, effector memory T cells; TEMRA, terminally differentiated effector memory CD45RA+.

**Supplemental Figure 4: Overall survival by CD8⁺ICOS⁺Ki67⁺ T-cell levels**. Kaplan–Meier curves showing overall survival in Cohort 1 stratified by CD8⁺ICOS⁺Ki67⁺ T-cell counts (MaxStat cutpoint: TP1 = 22).
